# Early Detection of Pancreatic Cancers Using Liquid Biopsies and Hierarchical Decision Structure

**DOI:** 10.1109/JTEHM.2022.3186836

**Published:** 2022-06-27

**Authors:** Deepesh Agarwal, Obdulia Covarrubias-Zambrano, Stefan H. Bossmann, Balasubramaniam Natarajan

**Affiliations:** Department of Electrical and Computer EngineeringKansas State University 5308 Manhattan KS 66506 USA; Department of Cancer BiologyThe University of Kansas Medical Center 21638 Kansas City KS 66160 USA

**Keywords:** Pancreatic cancer (PC), early cancer detection, liquid biopsy, information fusion, hierarchical decision structure

## Abstract

Objective: Pancreatic cancer (PC) is a silent killer, because its detection is difficult and to date no effective treatment has been developed. In the US, the current 5-year survival rate of 11%. Therefore, PC has to be detected as early as possible. Methods and procedures: In this work, we have combined the use of ultrasensitive nanobiosensors for protease/arginase detection with information fusion based hierarchical decision structure to detect PC at the localized stage by means of a simple Liquid Biopsy. The problem of early-stage detection of pancreatic cancer is modelled as a multi-class classification problem. We propose a Hard Hierarchical Decision Structure (HDS) along with appropriate feature engineering steps to improve the performance of conventional multi-class classification approaches. Further, a Soft Hierarchical Decision Structure (SDS) is developed to additionally provide confidences of predicted labels in the form of class probability values. These frameworks overcome the limitations of existing research studies that employ simple biostatistical tools and do not effectively exploit the information provided by ultrasensitive protease/arginase analyses. Results: The experimental results demonstrate that an overall mean classification accuracy of around 92% is obtained using the proposed approach, as opposed to 75% with conventional multi-class classification approaches. This illustrates that the proposed HDS framework outperforms traditional classification techniques for early-stage PC detection. Conclusion: Although this study is only based on 31 pancreatic cancer patients and a healthy control group of 48 human subjects, it has enabled combining Liquid Biopsies and Machine Learning methodologies to reach the goal of earliest PC detection. The provision of both decision labels (via HDS) as well as class probabilities (via SDS) helps clinicians identify instances where statistical model-based predictions lack confidence. This further aids in determining if more tests are required for better diagnosis. Such a strategy makes the output of our decision model more interpretable and can assist with the diagnostic procedure. Clinical impact: With further validation, the proposed framework can be employed as a decision support tool for the clinicians to help in detection of pancreatic cancer at early stages.

## I. Introduction

Pancreatic cancer (PC) is the third leading cause of cancer-related death in the US with a 5-year survival rate of 11% [1]. PC is characterized by a poor prognosis, invasiveness, rapid progression, and profound resistance to drug treatment, all which results in poor outcomes [2], [3]. Because of the virtual absence of early warning signs, PC is infrequently diagnosed at an early-stage [4], [5]. Consequently, neither surgical treatment, nor chemo- and radiotherapy are effective against PC [2], [3], [6], [7]. Currently, no universal screening tests for pancreatic cancer exists, and the best techniques available for pancreatic cancer detection are the commonly used ones, which include, biopsy and imaging test like endoscopic ultrasound, computerized tomography (CT) scans, magnetic resonance imaging (MRI), and positron emission tomography (PET) scans [8]. The medium survival rate of PC drops sharply with a later stage of detection. According to the American Cancer Society, the 5-year relative survival rate for PC is 39% at the localized state, 13% at the regional state, and 3% at the distant stage [1]. Therefore, a feasible and cost-effective Liquid Biopsy [9] for PC detection would be of great value, if it is capable of detecting PC at the localized stage, preferentially by means of a simple blood test. Liquid biopsies are of big interest for diseases like pancreatic cancer, where tissue samples are limited. Some liquid biopsies exploited for PC consist of identifying and quantifying tumor-associated components released from all tumor sources that can be present in blood, serum, or plasma, such as circulating tumor DNA (ctDNA), circulating tumor cells (CTCs), and extracellular vesicles (EVs) [10], [11]. Major difficulties with these liquid biopsy technologies include the lack in ability to isolate pure tumor-associated components, which typically contains a mix of tumor- and non-tumor associated components, which makes this technology insufficient to stand alone [11]. However, with the exception of the protease-activity technology discussed here, none of the “classic” approaches to Liquid Biopsies, such as the capture and detection of circulating tumor cell or circulating tumor DNA, DNA-methylation studies or the analysis of the content of extracellular vesicles, are capable of reliable detecting stage 1 PC [12]–[15], [16].

**FIGURE 1.**
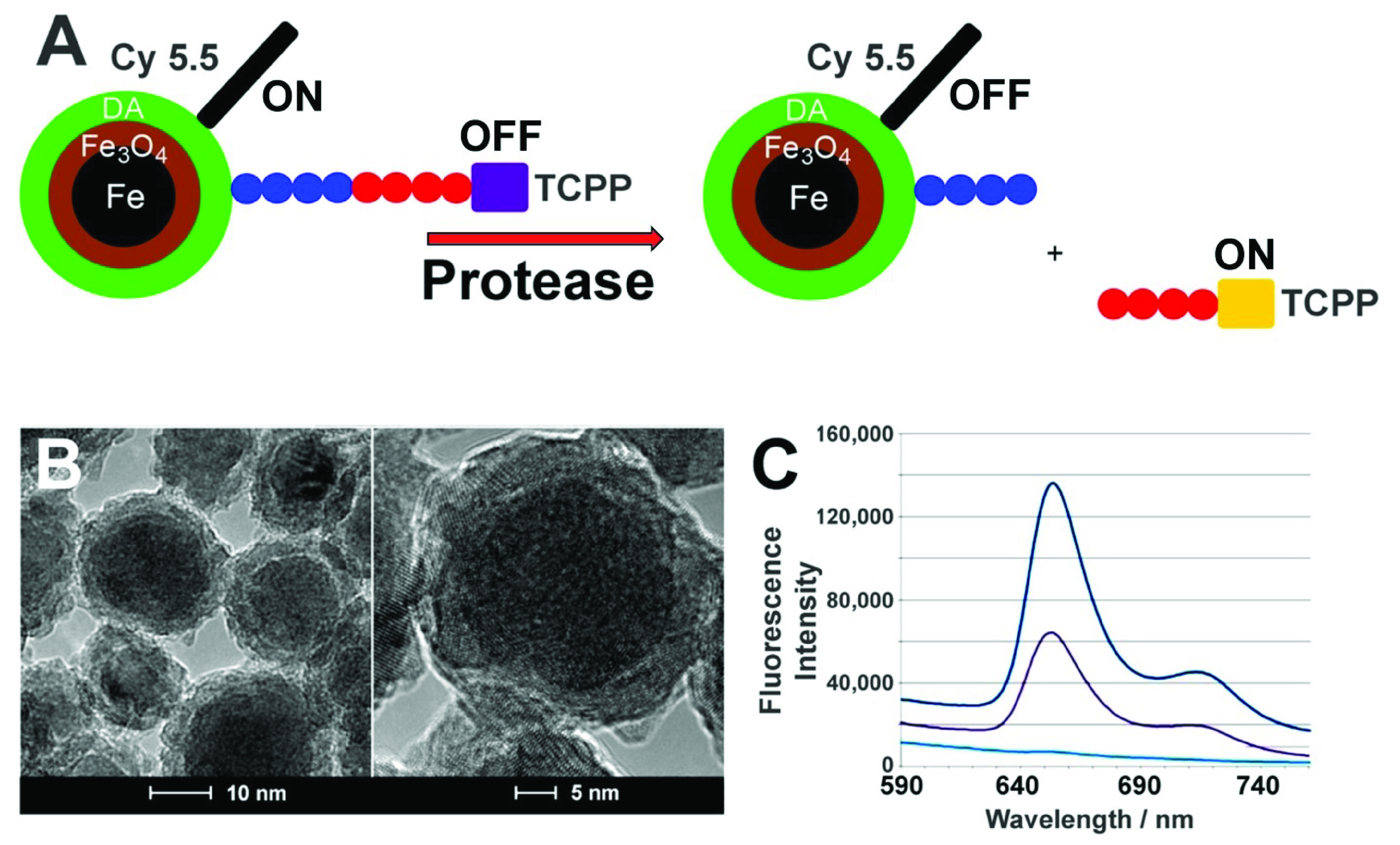
(A): Design principles of a nanobiosensor for protease detection. The OFF mode occurs when distance between fluorophore TCPP (tetrakis-carboxyphenyl-porphyrin), Fe/Fe_3_O_4_ nanoparticle, and FRET-acceptor cyanine (Cy) 5.5C is reduced, upon cleavage of the oligopeptide tether by a suitable protease present, this distance increases and leads to an increase in fluorescence intensity, which is called the ON mode. (B): TEM and HRTEM of dopamine-coated Fe/Fe_3_O_4_ core/shell nanoparticles. (C): Typical emission spectra occurring from a nanosensor for protease detection after 1h of incubation at 37 ^0^C ( $\lambda _{exc} = 421$ nm). low: buffer; middle: nanosensor; high: nanosensor after incubation with the respective enzyme; with permission from reference [22], copyright Elsevier, Amsterdam 2021.

The Bossmann group has established a panel of seven proteases (caspases B and E, matrix metalloproteinases (MMPs) 1, 3, and 9, urokinase plasminogen activator (UpA), and neutrophil elastase) and arginase as suitable panel of enzymes for early PC detection in 2018 [5]. This selection was based on Gene expression analysis [17] using data from NCBI GEO, Entrez Gene ID, Unigene ID and Gene Symbol [17]. Protease and Arginase activities in serum were measured with Fe/Fe_3_O_4_ core/shell nanobiosensors with an average particle size of 15 nm [5], [18]–[20], [21], [22]. The function principle is shown in Figure 1 (A). Each protease cleaves its respective consensus sequence and releases the fluorophore TCPP, which escapes the Förster quenching sphere of the nanoparticle plus tethered cyanine 5.5 dye (FRET pair) [18]. Upon escape, TCPP fluorescence increases and can be detected by a clinical plate reader. The fluorescence signal correlates with the fluorescence intensity [5]. Note that the nanobiosensor for arginase activity detection is not cleaved. Arginase performs a “post-translational” modification converting peptide-bound arginine into ornithine. The latter changes the dynamic of the peptide tether, which increases TCPP fluorescence [20].

Although statistically significant differences of the protease/arginase activity pattern of the group of all pancreatic cancer patients ( $n = 31$) and the group of healthy, age- and gender-matched volunteers ( $n = 48$) could be established utilizing these Fe/Fe_3_O_4_-based nanobiosensors, the overall sizes of the investigated groups was too small to establish the feasibility of early PC detection beyond reasonable doubt. Furthermore, simple biostatistics (e.g. performing Welch tests [23] and calculating p-values between data groups [23], [24]) do not provide the maximal extractable information from ultra-sensitive protease/arginase analyses. Therefore, we have employed information fusion based hierarchical decision structures for early-stage detection of pancreatic cancer. The classification models based on hierarchical decision structures are attracting significant research attention in the recent years. This is because they have demonstrated an appreciable predictive performance on a wide variety of interesting engineering applications like text classification [25], intrusion detection [26], manufacturing [27] and credit scoring prediction [28]. Biomedical applications like generation of molecular graphs [29], lung nodule malignancy classification [30], COVID-19 detection [31], skin lesion classification [32] and detection of Alzheimer’s disease [33] have also incorporated the use of hierarchical learning methods to build efficient classifiers. However, such hierarchical decision models have not been proposed for the early-stage detection of PC. Given the limited sample size in such studies, exploiting a hierarchical classification structure helps to reduce the complexity of model at each step, thereby opening the possibilities to improve the performance of traditional multi-class classification approaches. In this work, we propose a novel hierarchical framework for early-stage detection of pancreatic cancer. Firstly, a hard hierarchical decision structure (HDS) coupled with feature engineering at each step provides a better performance as compared to traditional multi-class classification approaches. Secondly, a soft hierarchical decision structure (SDS) additionally provides confidence associated with predicted labels in the form of probability values for each class.

The major purpose of using computational methods for early pancreatic cancer detection is detecting the onset of pancreatic cancer in the group of chronic pancreatitis patients, which would permit a maximal time for successful treatment with emerging methods, such as immunotherapy [34]. The key contributions of this work are as follows:

• This work, for the first time, proposes the use of ultrasensitive nanobiosensors for protease/arginase detection and integrates it with an information fusion based hierarchical decision structure to detect pancreatic cancer (PC) at the localized stage by means of a simple Liquid Biopsy.
• HDS, coupled with appropriate feature engineering steps is proposed to improve the performance of traditional multi-class classification approaches. Results illustrate up to 17% improvement in performance with the proposed HDS scheme relative to conventional multi-class classification approaches.
• To better assist the clinician’s decision-making and provide insights into the decision criteria driving the statistical methods, an SDS is developed to provide confidence scores associated with predicted labels, in the form of class probability values.

The decision labels and values of class probabilities obtained from HDS and SDS respectively support clinicians in recognizing situation where predictions of the computational models are uncertain. It helps them determine whether more tests are necessary for a more accurate diagnosis. In this manner, the proposed framework possesses the potential to serve as an effective decision support tool for early-stage PC detection. The remainder of this article is organized as follows: details pertaining to the dataset are presented in Section II, the proposed methodology is elucidated in Section III. The results are discussed in Section IV, followed by concluding remarks in Section V.

## II. Description of the Dataset

The dataset resulting from protease/arginase activity quantified using ultrasensitive nanobiosensors consists of a set of eight biomarkers. Identified biomarkers were obtained from the NCBI Gene expression omnibus (GEO) database, which is public accessible. Biomarkers were proteases with significant differences in expression levels between two samples, a primary tumor sample and a healthy tissue sample, which both had to be in *Homo sapiens*. The features for each sample comprise of values corresponding to a panel of seven proteases and arginase, selected based on gene expression analysis using data from Unigene ID, Gene Symbol, NCBI GEO and Entrez Gene ID [17]. Protease/arginase activity for each identified biomarker was quantified in human serum samples obtained from the Biospecimen Repository Facility in the Cancer Center of the University of Kansas Medical Center [35]. The group size was as follows: “Healthy” volunteers ( $n = 48$) and pancreatic cancer patients ( $n = 31$), which was further divided into “Localized” (earlier stage) and “Metastatic” (later stage) pancreatic cancer. Localized pancreatic cancer samples signify the absence of any indication that the cancer has spread outside the pancreas, while metastatic pancreatic cancer indicates that it has spread to other parts of the body as well. Quantified protease/arginase activity was quantified in serum samples after 60 min incubation, and this dataset was then utilized to develop a computer prediction model.

Although the sample size for this research study is limited, it is important to note that we are proposing a unique, one-of-a-kind approach for early cancer detection. We believe that providing these initial results will stimulate more follow-on efforts in this direction creating a significant clinical impact. Specifically, for this study, the team was not able to obtain more disease samples and had to work with the maximum number of samples the biospecimen repository at the University of Kansas Medical Center was able to recruit. It is imperative that the activity measured with this nanobiosensor technology depends strongly on the protocol and quality of the serum samples collected, which has been previously noticed by this team. For this reason, the better approach was to work with a smaller, but well-defined sample size instead of obtaining a larger sample size from different repositories to avoid multiple variables introduced during the comparison analysis. Furthermore, we provide confidence intervals for all our inferences in Section IV in order to accommodate the sample size effects and better illustrate the power of the results.

**FIGURE 2.**
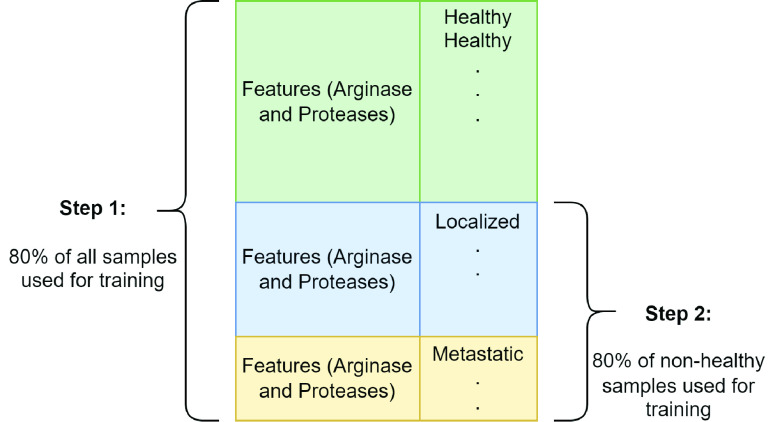
Formation of training set.

The training set formation process for individual binary classifiers at respective hierarchical steps is illustrated in Figure 2. Eighty percent of all the instances in the dataset are randomly selected to train the binary classifier in the first hierarchical step. This step results in the isolation of samples belonging to “healthy” group. So, 80% of the remaining instances, i.e., the samples from “localized” and “metastatic” groups are selected at random to form the training set for binary classifier in the second hierarchical step. This strategy for preparing the training sets for individual binary classifiers is adopted due to limited number of samples available in the dataset.

## III. Methods

In this work, the problem of early-stage detection of pancreatic cancer is modelled as a multi-class classification problem. The data derived from the experiments consists of four classes, namely, “Healthy”, “Localized” pancreatic cancer and “Metastatic” pancreatic cancer. Two information fusion based decision structures are proposed:

1) A HDS with specific feature engineering at each step for better performance relative to conventional classification approaches.
2) A SDS that provides confidence associated with predicted labels in the form of probability values for each class.

### A. Hard Hierarchical Decision Structure

**FIGURE 3.**
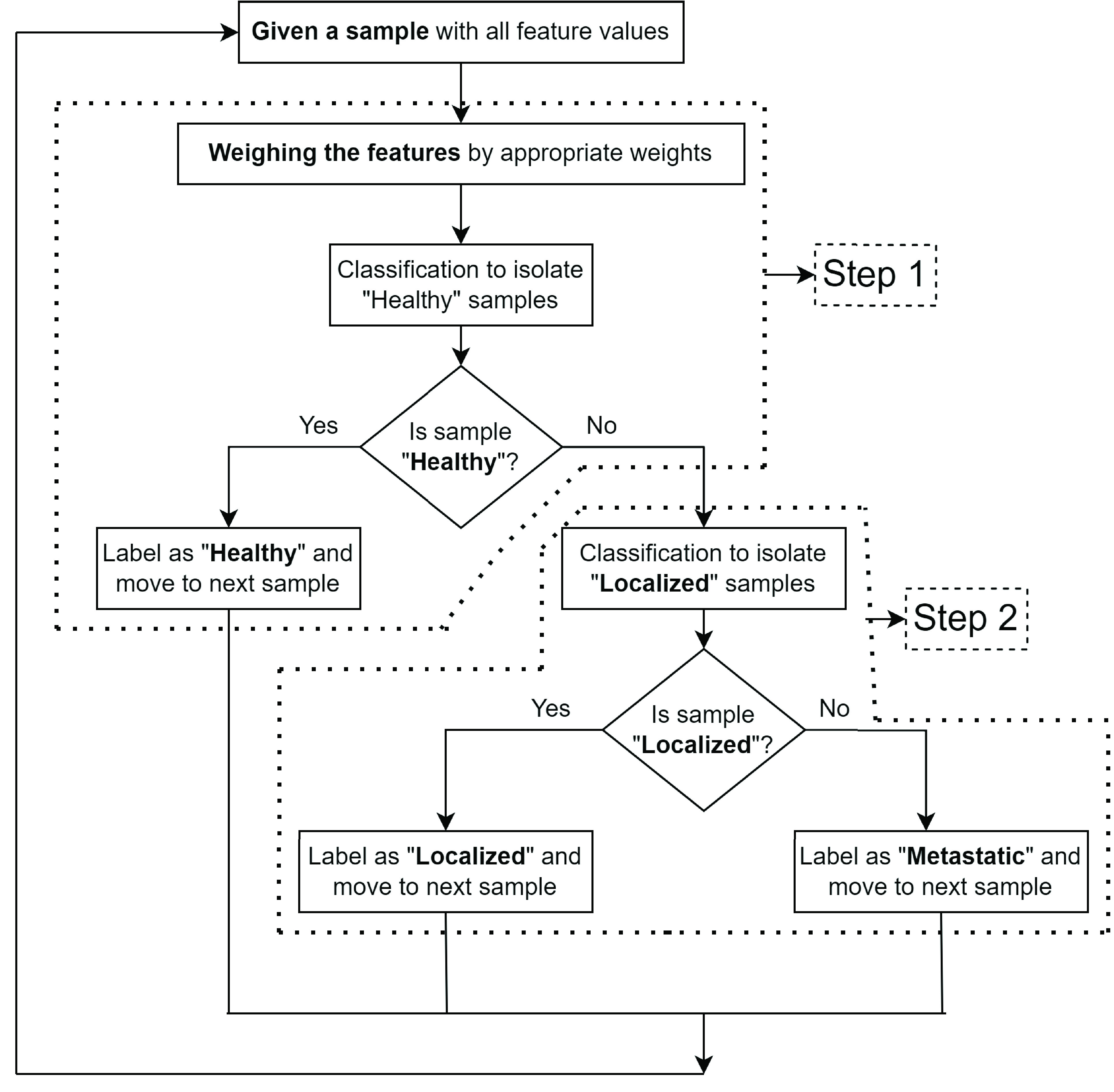
Proposed information fusion based HDS framework.

The fundamental premise of the proposed information fusion based HDS involves tailoring the statistically most significant features with appropriate weights to execute an efficient binary classification task at each hierarchical step. The proposed HDS is shown in Figure 3. The individual elements involved in building the HDS are described next.

#### 1) Computing Weights for Features

The first step in the proposed HDS framework is to identify whether the given sample belongs to healthy (null hypothesis) or non-healthy (alternate hypothesis) group. The feature engineering in building the corresponding binary classifier involves appropriate weighing of all the features based on their relative importance. These weights are obtained based on the $p$-values of two-sample t-tests for all the features across the set of measurements obtained from “healthy” and “non-healthy” groups. The test decision values and $p$-values for the null hypothesis that measures if “healthy” and “non-healthy” groups belong to independent random samples from normal distributions with equal means, were evaluated for all the features. It was observed that the $p$-values are distributed over a wide range and possess a highly skewed distribution. Therefore, the associated probability operations can generate extremely small values that are difficult to represent with sufficient precision. This results in numerical errors like underflow or overflow. In order to avoid precision issues, the $p$-values are transformed to a logarithmic scale for better interpretation and analysis [36]–[38]. The negative values of natural logarithm of $p$-values, $-\log _{e}p$ is computed for all the features and scaled, as shown in equation (1) to obtain the corresponding feature weights.

\begin{equation*} w_{i} = \frac {-\log _{e}f_{i}}{\sum _{n = 1}^{N} -\log _{e}f_{n}}\tag{1}\end{equation*}

Here, $w_{i}$ is the weight corresponding to feature $f_{i}$, $-\log _{e}f_{i}$ represents the negative value of natural logarithm of $p$-value corresponding to feature $f_{i}$ and $N$ is the total number of features. The $p$-values and computation of corresponding weights for all the features in the dataset under consideration are presented in Table 1.

**TABLE 1:** Computing Weights for the Features (A Test Decision Value of 0 Indicates a Failure to Reject the Null Hypothesis at 95% Confidence Level, a Value of 1 Indicates Rejection of the Null Hypothesis at 95% Confidence Level; A Rank of 1 Indicates That the Corresponding Feature is Most Important and That of 8 Indicates That the Corresponding Feature is Least Important)

Sr. No.	Feature	Test Decision	$p$-value	Rank	$-\log _{e}p$	Weight
1	Arginase	1	3.41e-5	6	10.29	0.0950
2	Cat B	1	8.92e-10	2	20.84	0.1925
3	Cat E	1	9.61e-10	3	20.76	0.1918
4	MMP 1	0	0.0538	8	2.92	0.0270
5	MMP 3	1	0.0214	7	3.84	0.0355
6	MMP 9	1	5.79e-10	1	21.27	0.1965
7	Neutrophil Elastase	1	1.62e-6	5	13.34	0.1232
8	UpA	1	3.02e-7	4	15.01	0.1387

#### 2) Selecting Features for Each Hierarchical Step

If a given sample is identified as “non-healthy” in the first hierarchical step, the next step is aimed at determining the degree or extent of abnormality involved. The second hierarchical step determines if the given sample belongs to a “localized” or “metastatic” group. The corresponding binary classifier uses a subset of the features rather than using all the features obtained from experiments, as in the first hierarchical step. This feature engineering step identifies the most relevant features, thereby simplifying the models and making them easier to interpret. Moreover, this allows to have shorter training times and reduces overfitting. The relevant features are identified by conducting a series of two-sample t-tests (as in the first hierarchical step) for localized vs. metastatic groups. The features exhibiting lowest $p$-values in hypothesis tests are selected as admissible features for corresponding binary classifier. For the dataset under consideration, Cat B, Cat E, MMP 3 and UpA are selected as admissible features for binary classifier in the second hierarchical step.

### B. Soft Hierarchical Decision Structure

**FIGURE 4.**
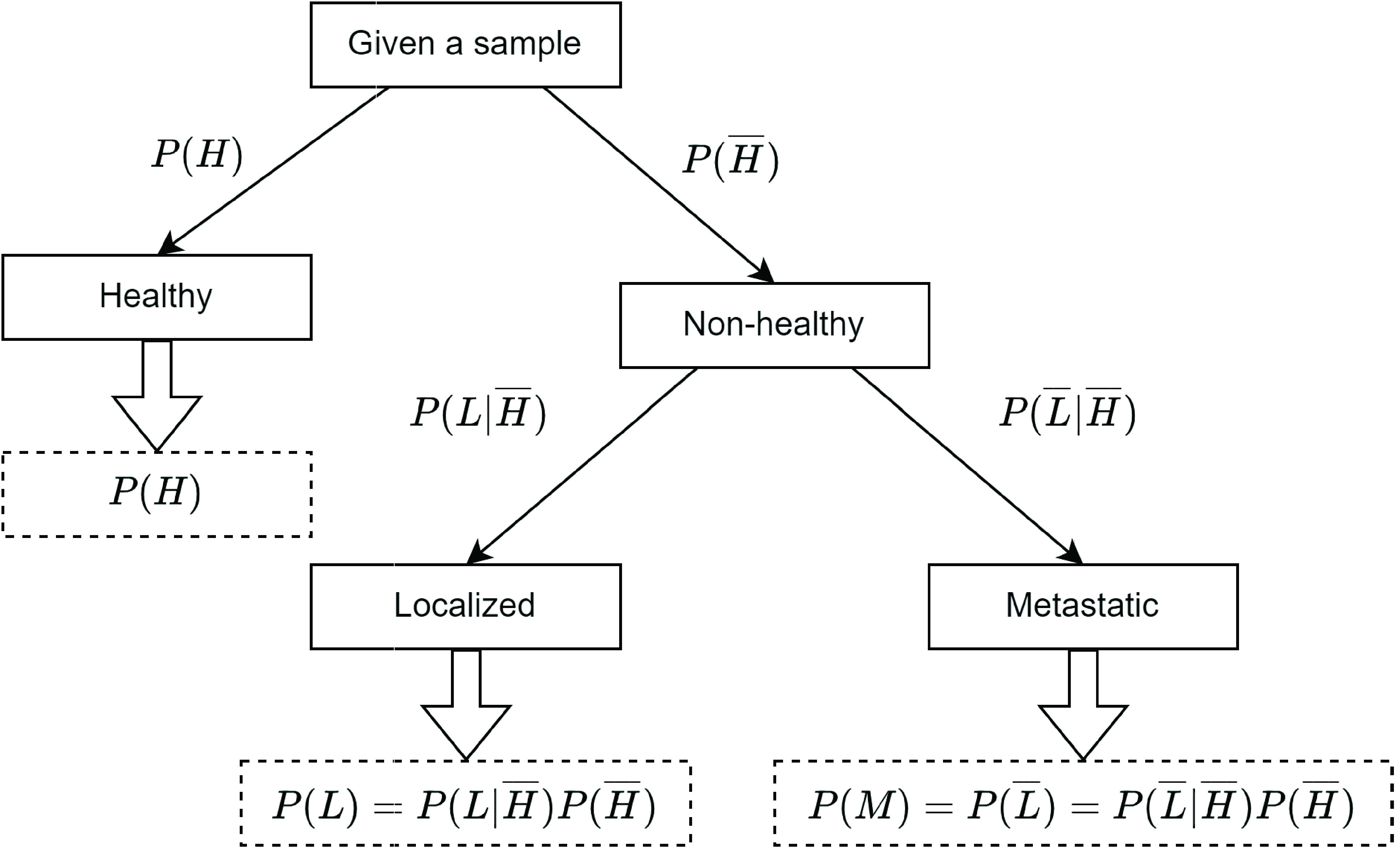
Proposed information fusion based SDS framework.

While the HDS offers a three-class classifier, it does not provide any information regarding the confidence associated with the decisions. This drawback is addressed in the proposed SDS framework that provides confidences associated with the predicted labels in the form of probability values for each class. The proposed SDS is shown in Figure 4. It is basically an extension of the HDS, where the prediction for each sample is accompanied with the probability values of that sample being affiliated to each of the three classes. The differences between these probability values provide an indication of confidence associated with the corresponding prediction. For a given instance, if the probability value corresponding to one of the classes in significantly higher than the rest, the confidence associated with such a prediction would be HIGH. On the other hand, if there is no significant difference between the probability values corresponding to all the classes, the associated confidence would be LOW. This framework helps the doctors determine whether additional tests are required for proper diagnosis.

All steps in the SDS are probabilistic extensions of the HDS. For example, the first step in SDS results in two values indicating probabilities of the given sample being “healthy” or “non-healthy”, represented by $P(H)$ and $P(\overline {H})$ respectively. These values are essentially the probability estimates of classification model trained at first hierarchical step for a given sample and are obtained using predict_proba() method of trained scikit-learn [39] models. The second hierarchical step evaluates the probabilities of the given sample being “localized” or “non-localized”, given the condition that it belongs to “non-healthy” group, represented by $P(L | \overline {H})$ and $P(\overline {L} | \overline {H})$ respectively. These values are probability estimates of classification model trained at second hierarchical step for a given sample. As a result, the probabilities of a sample being “localized” or “metastatic” is evaluated based on equations (2) and (3) respectively.

\begin{align*} P(L)=&P(L | \overline {H}) P(\overline {H}) \tag{2}\\ P(M)=&P(\overline {L}) = P(\overline {L} | \overline {H}) P(\overline {H})\tag{3}\end{align*}

## IV. Results and Discussion

### A. Hard Hierarchical Decision Structure

The proposed framework is evaluated by training a series of hierarchical classification models by considering several combinations of binary classifiers in all the three hierarchical steps, indicated in Figure 3. The classification methods considered for individual binary classifiers include: (i) Gaussian Naïve Bayes (GNB) [40], (ii) Decision Tree (DT) [41], (iii) Support Vector Machine (SVM) [42], (iv) k-Nearest Neighbors (kNN) [43], (v) Random Forest Classifier (RFC) [41], [43] and Logistic Regression (LR) [44]. In order to avoid overfitting, we have used $k$-fold ( $k = 5$) cross validation technique as a resampling method for training and evaluating the performance of classification models. The combinations of classification methods exhibiting an overall mean accuracy score of more than 85% are reported in Table 2. The sensitivity and specificity of all model combinations are also indicated. The 95% confidence intervals for evaluation metrics (accuracy score, sensitivity and specificity) are represented using mean and standard deviation of $k$-fold cross-validated values.

**TABLE 2:** Combinations of Classification Methods Exhibiting an Overall Mean Accuracy Score of More Than 85%

Sr. No.	Classification Method		Accuracy Score	Sensitivity	Specificity
	Step 1	Step 2			
1	GNB	DT	(87.34 ± 4.70)%	0.76 ± 0.10	0.84 ± 0.04
2	kNN	DT	(89.87 ± 6.28)%	0.81 ± 0.06	0.89 ± 0.06
3	kNN	kNN	(91.14 ± 3.94)%	0.85 ± 0.08	0.93 ± 0.04
4	kNN	SVM	(92.40 ± 3.26)%	0.93 ± 0.08	0.95 ± 0.06
5	RFC	DT	(88.61 ± 4.66)%	0.88 ± 0.04	0.91 ± 0.04
6	RFC	GNB	(86.07 ± 6.94)%	0.78 ± 0.06	0.88 ± 0.02
7	RFC	kNN	(89.87 ± 5.04)%	0.89 ± 0.02	0.86 ± 0.08
8	SVM	SVM	(88.61 ± 3.36)%	0.87 ± 0.08	0.90 ± 0.06
9	SVM	GNB	(87.48 ± 5.52)%	0.80 ± 0.06	0.88 ± 0.02
10	DT	GNB	(86.07 ± 7.76)%	0.78 ± 0.04	0.82 ± 0.08

The training sets were formed as described in Section II, and evaluation was performed over all the instances in the dataset under consideration. In Table 2, it can be observed that the best performance (overall mean accuracy score of 92.40%) is obtained using kNN for binary classification in first hierarchical step and SVM in the second step. Moreover, the sensitivity and specificity scores for this case are observed to the most favorable as compared to all other combinations of classification methods. The corresponding confusion matrix is presented in Table 3.

**TABLE 3:** Confusion Matrix – HDS (Step 1: kNN; Step 2: SVM)

		Predicted Class		
		Healthy	Localized	Metastatic
True Class	Healthy	46	1	1
	Localized	1	18	0
	Metastatic	1	2	9

On the contrary, the maximum mean classification accuracy obtained from conventional multi-class classification approach using individual classification methods is 74.66%, as indicated in Table 4. The 95% confidence intervals associated with the predictions of statistical models are reported using mean and standard deviation of $k$-fold ( $k = 5$) cross-validated accuracy scores. This demonstrates that the HDS framework outperforms the conventional multi-class classification approaches for early-stage detection of pancreatic cancer. The superior performance of proposed HDS framework is primarily attributed to the following reasons: (i) the features are weighed in the first hierarchical step based on their distinguishing ability, unlike traditional multi-class classification approaches which give equal importance to all the features; (ii) only a subset of features which are able to confidently differentiate between localized and metastatic PC are considered in the second hierarchical step, instead of accounting for all the features irrespective of their differentiating capability; (iii) splitting a multi-class classification problem into step-wise binary classification tasks allows for a more simplified feature representation and better learning.

**TABLE 4:** Performance Obtained Using Conventional Multi-Class Classification Approaches

Sr. No.	Classification Method	Accuracy Score
1	GNB	(70.48 ± 7.50)%
2	DT	(68.52 ± 11.22)%
3	SVM	(71.21 ± 5.74)%
4	kNN	(74.66 ± 4.28)%
5	RFC	(69.18 ± 7.82)%
6	LR	(72.95 ± 9.16)%

### B. Soft Hierarchical Decision Structure

**FIGURE 5.**
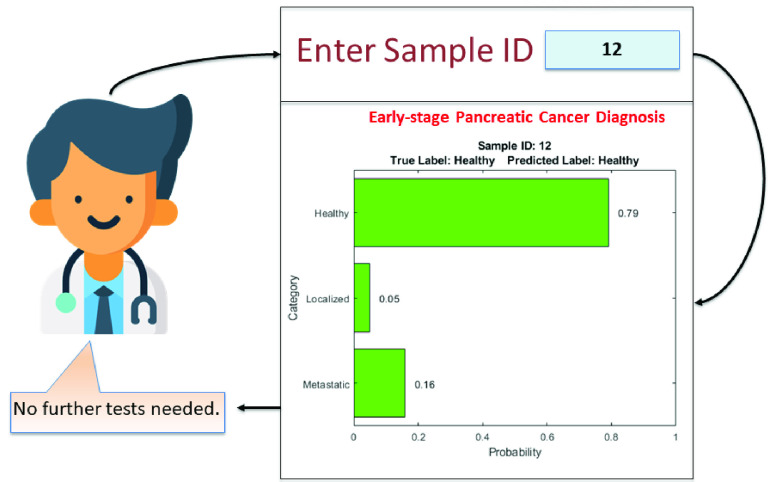
Example of correct prediction - SDS.

**FIGURE 6.**
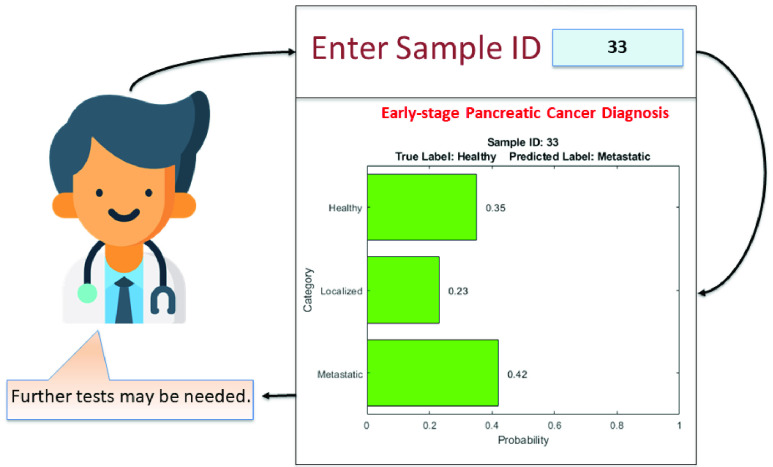
Example of incorrect prediction - SDS.

The proposed SDS framework supports computation of confidences associated with the predicted labels in the form of probability values for each class. An example for a correct and incorrect prediction are shown in Figure 5 and Figure 6 respectively.

The instance shown in Figure 5 is correctly classified as “Healthy”. It can be seen that the probability of this sample belonging to “Healthy” class is significantly higher than those of the other classes. In such a situation, the clinician can have sufficiently high confidence on the model prediction and it can be concluded that no further tests are required. In contrast, the instance shown in Figure 6 actually belongs to a “Healthy” class but is misclassified as “Metastatic”. Additionally, it can be observed that the differences in probabilities of “Healthy” and “Metastatic” classes is not as significant as in the instance demonstrated in Figure 5. One of the fundamental limitations of standard AI-based decision-making models is that they attempt to impose a strict conclusion in the form of an output by selecting the most appropriate option among all the possibilities. In such a scenario, the confidence and faithfulness towards predictions of these computational models is disputable, particularly for crucial applications such as medical diagnosis. The proposed SDS framework overcomes this shortcoming by specifying confidence associated with model predictions in the form of class probability values. This information helps the clinician perceive the lack of confidence in the model predictions and nudge them to possibly prescribe further tests prior to diagnosis. In a sense, the SDS builds off the HDS and makes it more “interpretable” to the end user.

## V. Conclusion

In this work, we have combined the use of ultrasensitive nanobiosensors for protease/arginase detection with information fusion based statistical framework to detect PC at the localized stage by means of a simple Liquid Biopsy. The information fusion based hierarchical decision structures are proposed for early-stage detection of pancreatic cancer. The HDS, coupled with feature engineering at each step exhibits an overall accuracy score of around 92%, as opposed to 74% obtained with conventional multi-class classification techniques. The SDS builds off the HDS to achieve a more “interpretable” outcome by providing confidence associated with predictions in terms of probability values for each class. This information can be used to clinicians in order to perceive the lack of confidence in model predictions and to examine if any further tests are required before making a final decision. The prime advantage of using such computational methods for detection of pancreatic cancer during early-stage is detecting the onset of pancreatic cancer in the group of chronic pancreatitis patients, which would allow a maximal time for successful treatment with emerging methods like immunotherapy.
